# Saccadic Eye Movements in Anorexia Nervosa

**DOI:** 10.1371/journal.pone.0152338

**Published:** 2016-03-24

**Authors:** Andrea Phillipou, Susan Lee Rossell, Caroline Gurvich, Matthew Edward Hughes, David Jonathan Castle, Richard Grant Nibbs, Larry Allen Abel

**Affiliations:** 1 Department of Optometry & Vision Sciences, The University of Melbourne, Melbourne, Victoria, Australia; 2 Department of Psychiatry, The University of Melbourne, Melbourne, Victoria, Australia; 3 Department of Mental Health, The Austin Hospital, Heidelberg, Victoria, Australia; 4 Brain and Psychological Sciences Research Centre, Swinburne University of Technology, Hawthorne, Victoria, Australia; 5 Monash Alfred Psychiatry Research Centre, Monash University, Clayton, Victoria, Australia; 6 Department of Psychiatry, St Vincent’s Hospital, Fitzroy, Victoria, Australia; 7 Faculty of Health Sciences, Australian Catholic University, Fitzroy, Victoria, Australia; UMR8194, FRANCE

## Abstract

**Background:**

Anorexia Nervosa (AN) has a mortality rate among the highest of any mental illness, though the factors involved in the condition remain unclear. Recently, the potential neurobiological underpinnings of the condition have become of increasing interest. Saccadic eye movement tasks have proven useful in our understanding of the neurobiology of some other psychiatric illnesses as they utilise known brain regions, but to date have not been examined in AN. The aim of this study was to investigate whether individuals with AN differ from healthy individuals in performance on a range of saccadic eye movements tasks.

**Methods:**

24 females with AN and 25 healthy individuals matched for age, gender and premorbid intelligence participated in the study. Participants were required to undergo memory-guided and self-paced saccade tasks, and an interleaved prosaccade/antisaccade/no-go saccade task while undergoing functional magnetic resonance imaging (fMRI).

**Results:**

AN participants were found to make prosaccades of significantly shorter latency than healthy controls. AN participants also made an increased number of inhibitory errors on the memory-guided saccade task. Groups did not significantly differ in antisaccade, no-go saccade or self-paced saccade performance, or fMRI findings.

**Discussion:**

The results suggest a potential role of GABA in the superior colliculus in the psychopathology of AN.

## Introduction

Anorexia Nervosa (AN) is a psychiatric illness characterised by three main criteria: significantly low body weight, a fear of weight gain and a disturbance in the experience of one’s own body weight or shape [1]. AN has a mortality rate among the highest of any mental illness [2,3], though the causal and maintenance factors of the condition remain unclear. A number of neurobiological findings have been reported in AN employing a range of neuroimaging techniques, though, these are highly inconsistent (for a review see [4]). An alternative tool that could be used to investigate neurobiological differences between AN and healthy control groups is eyetracking. Eye movements, particularly saccadic eye movements (rapid shifts of gaze), are controlled by known brain circuits, cortical and subcortical. Furthermore, poor performance on a range of saccadic tasks has been reported in a variety of psychiatric conditions. Yet, to date, saccadic eye movements have not been systematically investigated in AN.

Prior to examining the relevant literature, a brief description of the types of saccadic tasks and what they tell us about eye movements and cognitive processing is pertinent. The *prosaccade task* is the simplest saccade task since it merely requires a visually-guided saccade to a sudden onset peripheral stimulus. The *antisaccade task* requires a voluntary saccade to the opposite direction of a stimulus, and is a sensitive measure of inhibitory control. The *no-go saccade task* is also a measure of inhibitory control as participants are required to remain fixated on a central stimulus and not make a saccade toward a peripherally presented stimulus. The *memory-guided saccade task* requires participants to generate a saccade toward a remembered location following a brief delay, and is not only a measure of short term memory, but also of inhibition as participants are required to refrain from making a saccade toward the remembered location until a cue is presented. The *self-paced saccade task* on the other hand is considered to be an almost entirely volitional task as no cues are presented to guide the constant refixations from one target to another.

These saccade tasks have been utilised to elucidate deficits in a variety of psychiatric conditions. In schizophrenia, an increased rate of inhibitory errors, increased latencies and reduced accuracy of correct responses has been consistently found on the antisaccade and memory-guided saccade tasks (e.g. [5,6]). Increased intraindividual variability of intersaccadic intervals has also been found for the self-paced saccade task [7], though performance on prosaccade tasks is typically intact in schizophrenia patients (e.g. [8,9]). Of particular relevance to AN are the findings in obsessive compulsive disorder (OCD) as the condition often occurs comorbidly in AN and the two conditions share similar symptom expressions [10]. Similarly to the findings in schizophrenia, individuals with OCD have consistently been found to display intact prosaccade performance [11–15]. The findings related to antisaccade performance are, however, rather inconsistent. Several studies have reported no difference between OCD and healthy controls on antisaccade errors [12,14–17] or latency [13,14,16]. Other studies have reported both increased antisaccade latency [12,15,17] and errors [11,13,18], particularly for targets of smaller amplitude [13,19]. Additionally, patients with OCD have been found to not differ in error rate on a no-go saccade task [15], but to make more suppression errors on a memory-guided saccade task [13].

Many studies have investigated the neural basis of saccade performance using the blood oxygen level dependent (BOLD) technique with functional magnetic resonance imaging (fMRI). Activity associated with antisaccade performance has been of particular research interest. In schizophrenia, studies have typically compared blocks of antisaccades to prosaccades between groups and reported reduced activity in the frontal and supplementary eye fields (FEF; SEF), and the dorsolateral prefrontal cortex (DLPFC) [9,20]. However, employing blocked designs results in activity related to both preparatory periods and saccade execution, which have been found to result in different activations when examined separately. Increased activity to antisaccades relative to prosaccades has been reported in frontal areas of the brain, including the FEF and DLPFC, during preparatory periods, but no difference in activity during response periods [21,22]. Brown et al. [23] on the other hand utilised an interleaved prosaccade/antisaccade/no-go (PAN) saccade task and found no difference in activity during preparatory periods between conditions. However, there was increased activity in the FEF, SEF, anterior cingulate cortex (ACC), precuneus and intraparietal sulcus during antisaccade responses relative to prosaccade and no-go responses. The discrepancy in findings likely reflects differences in methodology between studies, and may be related to the extended preparatory and response period epochs in the earlier two studies compared to that of Brown et al. [23].

To our knowledge, saccadic eye movements have not hitherto been examined in any eating disorder, including AN. Examining saccadic eye movements performance in AN will allow not only for the investigation of similarities in reported performance to related conditions, such as OCD, but also has the potential to demonstrate brain areas and neurotransmitter systems of possible dysfunction in AN. Therefore, the aim of this study was to investigate performance on a battery of saccadic eye movement tasks between individuals with AN and healthy controls. Participants were presented with a PAN saccade task during fMRI and concurrent eyetracking, and a memory-guided saccade and self-paced saccade task behaviourally without functional neuroimaging. Building on findings in OCD (see above), we hypothesised that AN participants would show poorer performance on the antisaccade and no-go components of the PAN task than controls, as demonstrated by an increased rate of antisaccade and no-go errors, and increased latency and reduced gain of correctly executed antisaccades. Prosaccade performance was expected to be intact. Related to the hypothesised performance on the PAN task, the AN group were hypothesised to show reduced activity in the FEF, SEF, ACC and DLPFC during correct antisaccade response periods, compared to prosaccades and no-go response periods, but to not differ from healthy individuals during preparatory periods. Poorer performance on the memory-guided saccade task was also expected in the AN group, evident by an increased rate of inhibitory errors, and increased latency and reduced gain of correct responses. Self-paced saccade performance was not expected to differ between groups.

## Materials & Methods

This study was approved by the human research ethics committees at The University of Melbourne, Swinburne University of Technology, The Melbourne Clinic, The Austin Hospital and St Vincent’s Hospital; all in Melbourne, Australia. Informed written consent was obtained from all participants. All procedures contributing to this work comply with the ethical standards of the relevant national and institutional committees on human experimentation and with the Helsinki Declaration of 1975, as revised in 2008.

### Participants

Twenty-four right-handed individuals with AN and 25 healthy control individuals (HCs) completed the behavioural eyetracking tasks (memory-guided saccades and self-paced saccades tasks), and the PAN saccade task in the MRI scanner. Technical eyetracking difficulties in the *MRI* resulted in the data of three HCs being excluded, allowing analyses to be conducted on 24 AN and 22 HC participants. Technical eyetracking difficulties in the *behavioural* set up resulted in the data of one HC being excluded, such that the analyses presented here are on 24 AN and 24 HC participants. HCs were recruited through public advertisements, whereas AN participants were recruited through public advertisements, the Body Image and Eating Disorders Treatment and Recovery Service at the Austin and St Vincent’s Hospitals, and The Melbourne Clinic; all in Melbourne, Australia.

All participants were English speaking, had no history of significant brain injury or neurological condition, no significant ocular pathology and normal (or corrected to normal) visual acuity. HCs were required to have no history of an eating disorder or other mental illness; they were also required to not be taking any medications apart from hormonal contraceptives (11 HC participants were taking this medication). AN participants were instructed to continue with their normal medications, which were: selective serotonin reuptake inhibitors (SSRIs) (10), atypical antipsychotics (10), benzodiazepines (5), serotonin-noradrenaline reuptake inhibitors (SNRIs) (3), hormonal contraceptives (3), melatonergic antidepressants (2), noradrenergic and specific serotonergic antidepressant (NaSSA) (1) and cyclopyrrolones (1). None of the participants were smokers.

The Mini International Neuropsychiatric Interview, 5.0.0 (MINI) [24] was used to screen participants for Axis I psychiatric disorders according to the Diagnostic and Statistical Manual of Mental Disorders (DSM-IV). It was also used to confirm diagnoses of AN, with the exception of the amenorrhea criterion which is no longer included in the current DSM-5. AN was required to be the primary diagnosis of the AN group. AN participants with comorbid psychiatric conditions, other than psychotic conditions, were not excluded as this would not have represented a typical AN sample.

Premorbid intelligence was estimated using the Wechsler Test of Adult Reading (WTAR) [25]. Eating disorder symptomatology was investigated with the Eating Disorders Examination Questionnaire (EDE-Q) [26] (Table 1).

**Table 1:** Participant information.

	AN		HC		
	M	SD	M	SD	*p*
Age	23.07	6.88	22.67	3.19	0.798
Premorbid IQ	104.67	8.19	105.60	7.00	0.670
BMI	16.52	1.14	22.40	3.59	0.001
Illness duration	6.67	7.66	-	-	-
Age of illness onset	16.04	3.50	-	-	-
EDE-Q restraint	3.93	1.42	0.58	0.63	0.001
EDE-Q eating concern	3.78	1.24	0.25	0.31	0.001
EDE-Q shape concern	5.01	0.90	1.17	0.84	0.001
EDE-Q weight concern	4.50	1.41	0.66	0.82	0.001
EDE-Q global score	4.30	1.12	0.67	0.54	0.001

Note: AN = Anorexia Nervosa; HC = healthy controls; Premorbid IQ = standardised Wechsler Test of Adult Reading Score; BMI = body mass index; EDE-Q = Eating Disorders Examination Questionnaire; Age, age of illness onset and duration illness are reported in years

### Tasks

#### Self-paced saccades

Participants were presented with two 1° dots at ±10° from the centre of the monitor for 30 seconds and were asked to fixate from one dot to the other as fast as they were able to, for the entire duration of the task. Prior to the presentation of the peripheral stimuli, a fixation cross was presented in the centre of the monitor so that all participants had the same starting point. The number of saccades was counted, and the gain (ratio between primary saccade and target amplitude), intersasccadic interval (interval between saccade *onsets*) and peak saccadic velocity of saccades were calculated. Catch-up saccades were deleted and only the primary saccade was analysed. In the case of multiple saccades in the same direction before a saccade in the opposite direction, the time between primary saccades was not analysed.

#### Memory-guided saccades

Participants were presented with a 1° fixation cross in the centre of the computer monitor for a pseudorandom period (1000–3500ms) prior to the onset of a peripheral stimulus for 50ms. The peripheral stimulus was presented in a pseudorandom location (5° or 10° to the left or right) while participants were required to continue fixating on the central stimulus. Following the presentation of the peripheral stimulus, the central stimulus remained on screen for a further pseudorandom period (1000–3500ms), prior to its offset when a blank screen appeared for 1000ms. Only once this blank screen was presented were participants required to make a saccade to the location of the briefly presented peripheral stimulus. Participants were presented with a total of 52 trials, with an equal number of target presentations to each peripheral location. Participants also completed a practice task consisting of five trials prior to the commencement of the task. The task was analysed for percentage of inhibitory/anticipation errors, percentage of directional errors and percentage of correct responses for 5° and 10° stimuli separately and together. Inhibitory errors were classified as saccades greater than 2° toward the stimulus that were made between the 50ms stimulus onset and the beginning of the response period, or within 80ms of the response period. The gain and latency of correct saccades were also calculated, as was the peak saccadic velocity for 5° and 10° saccades separately. Correct responses were only analysed if the participant was fixated on the central cross prior to and following the appearance of the peripheral dot. Saccades smaller than 2° were not analysed.

#### Prosaccade/antisaccade/no-go saccade task

Participants were initially presented with a 1° fixation cross for a period of 2000ms. The central fixation cross was then replaced by a coloured dot acting as the cue period for a pseudorandom period (2700–3500ms). If the dot was green, it indicated that a prosaccade was required in the upcoming trial (a saccade to the stimulus); if the dot was blue it indicated that an antisaccade was required (an inhibited response to the peripheral stimulus, and a saccade to its mirror image); and if the dot was red it indicated a no-go response (to inhibit the response to the peripheral stimulus and remain fixated on the centre of the screen). Targets were presented pseudorandomly at 5° and 10° to the left and right in a step-wise fashion (i.e. not gap or overlap paradigms). The response period varied pseudorandomly between 2000–2800ms and participants were required to continue fixating on the correct location until the central fixation cross reappeared. The PAN task was split up into three runs, with 18 presentations of each stimulus type in each run, resulting in a total of 54 trials per stimulus type, with an equal number of presentations at each target location. Prior to entering the scanner, participants completed a practice task consisting of two trials per stimulus type. Each stimulus type was analysed for percent errors (prosaccade error = saccade in the opposite direction of the stimulus; antisaccade error = saccade to the stimulus; no-go error = saccade to the stimulus), gain, latency, and peak saccadic velocity (5° and 10° targets separately) of correct responses. Antisaccade errors were also separated into corrected and uncorrected errors and analysed separately. Saccades smaller than 2° were not analysed.

### Data acquisition and analysis

#### Eyetracking

Stimuli were presented through SR Research’s Experiment Builder program, and eye movements were recorded using a remote view eyetracker, the EyeLink1000 (SR Research, Ontario, Canada), monocularly at 500Hz. Data were analysed with SR Research’s analysis program, DataViewer. The memory-guided and self-paced saccade tasks were presented on a 17” screen with a resolution of 1024x768, 90cm away from the participant who rested on a chinrest. The PAN task was presented while participants underwent an fMRI scan on a 24” monitor at the rear of the bore, 115cm from the participant’s eye.

Saccades were detected using the EyeLink1000 system’s saccade detection algorithm: a saccade velocity threshold of 30°/sec, an acceleration threshold of 8000°/sec^2^ and a motion threshold of 0.15°.

#### fMRI

MRI scans were undertaken with the Siemens Tim Trio 3 tesla system with a 32 channel head coil at Swinburne University of Technology (Melbourne, Australia). During each functional run of active task performance, 612 T2*-weighted images were acquired oblique to the commissural plain using an interleaved multiband sequence (multiband acceleration factor = 4, bandwidth = 25988 Hz/Px, repetition time (TR) = 710ms, echo time (TE) = 30ms, echo spacing = 0.51ms, flip angle = 52°, field of view = 222mm, voxel resolution = 3x3x3mm, slice thickness = 3mm, number of slices = 44). Multiband acquisition sequences were derived from the Human Connectome Project [27]. A T1-weighted image was acquired sagitally for anatomical reference (bandwidth = 170 Hz/Px, TR = 1900ms, TE = 2.52ms, echo spacing = 7.5ms, flip angle = 9°, field of view = 256mm, voxel resolution = 1x1x1mm, slice thickness = 1mm).

MRI data pre-processing and statistical analyses were performed using SPM8, through Matlab R2014a (Mathworks, Natick, MA, USA). Image pre-processing included image realignment, then coregistration of the T1 image to a mean realigned functional image created during realignment. The co-registered T1 image was then normalised to the T1 template supplied with SPM8 (Montreal Neuroimaging Institute, MNI), then the parameters of this transformation were applied to realigned functional images. The normalised functional images were then spatially smoothed with a Gaussian kernel of 8x8x8mm.

### Statistical analyses

#### Behavioural and eyetracking analyses

Performance on eyetracking components was compared with between groups analyses of variance (ANOVAs), following normality checking and the removal of outliers (less than one outlier per group, on average). Continuous variables which violated the assumptions of an ANOVA were analysed with Mann-Whitney U tests. SPSS version 21 was used, with alpha set at .05 for all analyses. Pearson’s correlation analyses were also performed between EDE-Q scores and the eyetracking data (no significant findings).

#### fMRI analyses

First-level modelling was performed by fitting a convolved hemodynamic response function (HRF) and its temporal derivative separately to the onset times of correct prosaccade, antisaccade and no-go responses. Errors on each trial type were modelled together, as were missing trials and anticipatory responses for each trial type. Due to the small number of errors made for each group, only the correct responses were contrasted. As discussed, cue and response periods can result in different activations and it is therefore more appropriate to model them separately, therefore resulting in six regressors plus their temporal derivative. After parameter estimation, the following six contrast images were produced as in Brown et al. [23]: antisaccade cue > no-go cue, antisaccade cue > prosaccade cue, prosaccade cue > no-go cue, antisaccade response > no-go response, antisaccade response > prosaccade response, and prosaccade response > no-go response.

At the group level, these contrast images were first entered into one-way ANOVA models for AN and HC groups separately to investigate within-group effects. Group differences were interrogated with a mixed-effects ANOVA model using the flexible factorial option in SPM 8. This model included a between-subjects *group* factor (2 levels: patients vs controls), a within subjects *condition* factor (6 levels: antisaccade cue > no-go cue, antisaccade cue > prosaccade cue, etc.) and a *subjects* factor (number of levels equals the number of participants) that controlled for within-subject variability [28].

T-statistic images were corrected for multiple comparisons using the random-field theory approach at the voxel and cluster levels (p < .05, FWE-corrected). For the one-way within groups ANOVAs, the main effect of condition and simple effects for each condition were analysed for each group. The mixed-design analysis involved the investigation of a group by condition interaction, followed by simple effects comparing each condition between groups.

## Results

### Self-paced saccade task

Groups were not found to significantly differ on any component of the self-paced saccade task, though there was a trend for AN participants to show a longer average intersaccadic interval (Table 2). Further analyses were performed comparing the variability between groups for each measure, though none of the analyses resulted in significant group differences.

**Table 2:** Self-paced saccade task results.

	AN		HC				
	M	SD	M	SD	F	*p*	*Cohen’s d*
Saccade rate	67.50	14.41	72.50	9.28	2.04	0.160	0.41
Gain	0.90	0.17	0.89	0.07	0.05	0.831	0.06
Intersaccadic interval	464.46	156.12	401.67	51.28	3.50	0.068	0.54
Peak velocity	446.74	191.18	455.16	63.41	0.04	0.839	0.06

Note: AN = Anorexia Nervosa; HC = healthy controls; saccade rate is the number of saccades made in 30 seconds; intersaccadic interval is reported in milliseconds; peak velocity is reported in degrees/second

### Memory-guided saccade task

**Fig 1:**
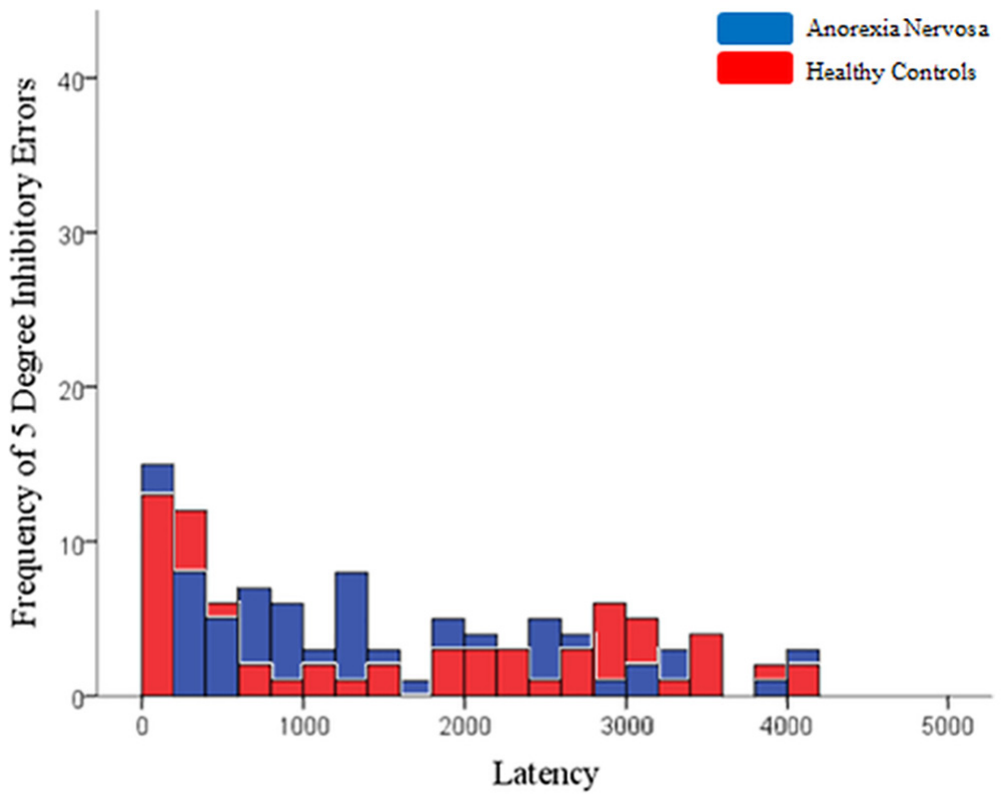
Frequency of individual memory guided-saccades inhibitory error latencies at 5° targets, within 200 millisecond bins.

**Fig 2:**
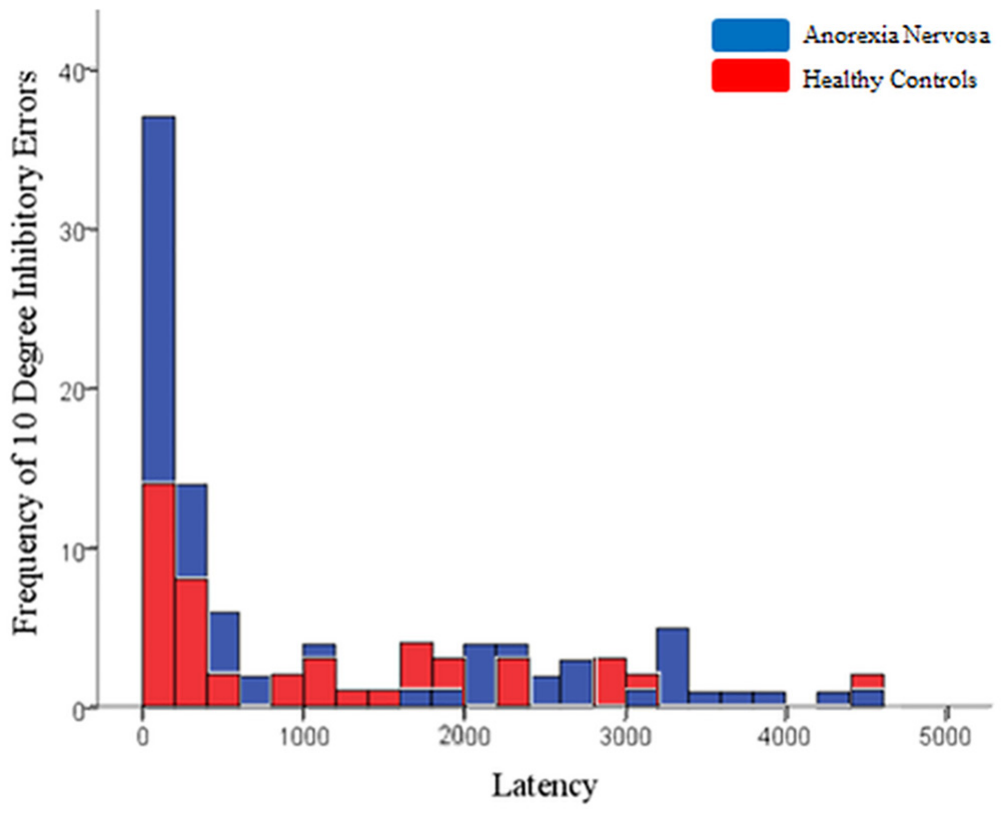
Frequency of individual memory guided-saccades inhibitory error latencies at 10° targets, within 200 millisecond bins.

On the memory-guided saccade task, groups differed significantly on inhibitory error rate for 10° targets, with AN participants making more inhibitory errors (*F*(1,42) = 10.444, *p* = .002, *Cohen’s d* = .993). Groups did not differ significantly on any other component of the memory-guided saccade task, though the latency of correct memory-guided saccades fell just short of significance, indicating a trend for AN participants to display longer latencies (Table 3). The distribution of latencies for individual inhibitory errors at 5° and 10° target distances are displayed in Figs 1 and 2. The distributions suggest that AN participants made more inhibitory errors of shorter latency to 10° targets in the range of reflexive saccade latencies, though the medians did not significantly between groups (*U*(792) = 71880.50, *Z* = -.630, *p* = .529).

**Table 3:** Memory-guided saccade task results.

	AN		HC				
	M	SD	M	SD	*F*	*p*	*Cohen’s d*
Gain	0.78	0.08	0.78	0.06	0.04	0.852	0.01
Latency	296.98	34.56	277.26	32.90	3.84	0.057	0.58
Peak velocity, 5°	166.72	30.51	167.95	25.39	0.02	0.882	0.04
Peak velocity, 10°	248.07	38.53	265.70	26.64	3.30	0.076	0.53
Inhibitory error rate	14.81	10.98	8.47	5.32	5.77	0.021	0.74
5°	14.55	10.24	11.54	7.50	1.22	0.276	0.34
10°	16.22	13.52	6.04	5.24	10.44	0.002	0.99
Directional error rate	0.24	0.86	0.16	0.54	0.15	0.702	0.11

Note: AN = Anorexia Nervosa; HC = healthy controls; gain, latency and peak velocity are reported for correct responses only; latency is reported in milliseconds; peak velocity is reported in degrees/second

### Prosaccade/antisaccade/no-go saccade task

#### Eyetracking

**Fig 3:**
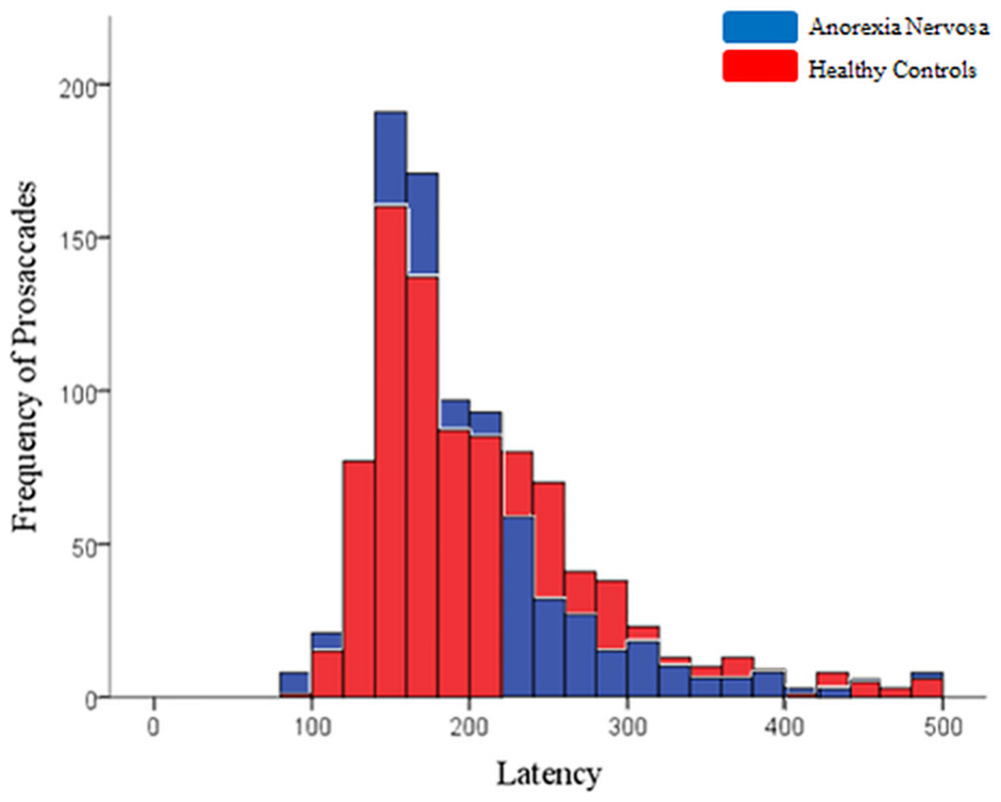
Frequency of individual prosaccade latencies within 20 millisecond bins.

Groups did not significantly differ in error rate on any trial type of the PAN task as presented in Table 4. The saccade characteristics of correct prosaccade and antisaccade trials are presented in Table 5. The latency of correct prosaccades made in the PAN task was found to be shorter in AN participants (*F*(1,43) = 7.01, *p* = .011, *Cohen’s d* = -.784). Groups were not found to significantly differ on any other component of prosaccade trials. The distribution of latencies for individual prosaccades also did not indicate an increased rate of express saccades in AN (i.e. saccades made between 80–120ms) (Fig 3).

**Table 4:** Prosaccade/antisaccade/no-go saccade task error rates.

	AN		HC				
	M	SD	M	SD	*F*	*p*	*Cohen’s d*
Prosaccade	1.39	2.58	1.43	1.61	0.01	0.948	0.02
Antisaccade	18.75	12.72	16.16	11.05	0.54	0.467	0.22
*Corrected*	18.13	12.34	15.82	10.75	0.45	0.504	0.20
*Uncorrected*	0.62	1.61	0.34	0.93	0.51	0.478	0.21
No-go	14.51	11.27	12.58	7.98	0.44	0.511	0.20
Total	11.55	7.18	10.06	4.97	0.66	0.422	0.24

Note: AN = Anorexia Nervosa; HC = healthy controls; response rates reported as percentages.

**Table 5:** Saccade characteristics of correct prosaccade and antisaccade responses.

	AN		HC				
	M	SD	M	SD	*F*	*p*	*Cohen’s d*
Prosaccades							
* Gain*	0.91	0.09	0.92	0.04	0.03	0.875	0.14
* Latency*	206.87	33.72	250.42	70.97	7.01	0.011	0.78
* Peak Velocity 5*°	209.36	37.47	211.06	32.32	0.03	0.870	0.05
* Peak Velocity 10°*	288.34	43.76	286.90	35.00	0.02	0.903	0.04
Antisaccades							
* Gain*	0.95	0.22	0.86	0.16	2.39	0.129	0.47
* Latency*	333.00	82.35	364.78	85.72	1.64	0.207	0.38
* Peak Velocity 5*°	225.01	55.12	220.04	49.28	0.10	0.752	0.10
* Peak Velocity 10°*	238.47	56.21	247.63	56.18	0.31	0.584	0.16

Note: AN = Anorexia Nervosa; HC = healthy controls; latency is reported in milliseconds; peak velocity is reported in degrees/second for 5° and 10° targets separately

#### fMRI

*One-way within groups ANOVAs*: For the *AN group*, a significant main effect of condition was found. Simple effects are presented in Table 6. The antisaccade cue > no-go cue contrast resulted in one significant cluster covering the bilateral cuneus, calcarine sulcus, primary visual cortex (V1) and secondary visual cortex (V2). The antisaccade cue > prosaccade cue contrasts revealed two significant clusters: one in the right middle and superior frontal gyri, and one in the left middle and superior frontal gyri. The prosaccade cue > no-go cue resulted in increased activation in one cluster in the bilateral cuneus, calcarine sulcus, V2, V1, posterior cingulate cortex and associative visual cortex.

**Table 6:** Significant within groups contrasts for the Prosaccade/Antisaccade/No-Go task for Anorexia Nervosa participants.

Contrast	No. of voxels	Peak *t*	Peak MNI coordinates			Peak regions
			x		z	
*Cue period*						
Antisaccade > no-go	776	6.60	14	-84	8	Cuneus, calcarine
						sulcus, V1, V2
Antisaccade > prosaccade	128	5.50	24	-8	54	Middle & superior
						frontal gyri
	80	5.40	-24	-10	58	Middle & superior
						frontal gyri
Prosaccade > no-go	2592	8.69	-10	-86	12	Cuneus, calcarine
						sulcus, V2, V1,
						posterior cingulate
						cortex, associative
						visual cortex
*Response period*						
Antisaccade > no-go	126	6.95	-8	8	54	SMA, medial &
						superior frontal
						gyri, premotor cortex
	104	5.50	-25	-2	52	Middle frontal gyrus
Antisaccade > prosaccade	240	6.46	-8	8	54	SMA, medial &
						superior frontal gyri,
						premotor cortex
Prosaccade > no-go	81	8.45	28	-98	-4	Inferior & middle
						occipital gyrus

Note: V1 = primary visual cortex; V2 = secondary visual cortex; SMA = supplementary motor area: MNI = Montreal Neuroimaging Institute

The antisaccade response > no-go response contrast resulted in two significant clusters: the first in the left supplementary motor area (SMA), medial frontal gyrus, superior frontal gyrus, premotor cortex, and the second in the left middle frontal gyrus. The antisaccade response > prosaccade response contrasts resulted in a significant cluster covering the same areas as the first cluster of the antisaccade response > no-go response contrast described above. Finally, the prosaccade response > no-go response contrast resulted in increased activity in one cluster in the right inferior and middle occipital gyrus.

For the *HCs*, a significant main effect of condition was also found. Individual contrasts for the control group are presented in Table 7. The antisaccade cue > no-go cue contrast resulted in increased activity in two clusters: a cluster in the bilateral cuneus, calcarine sulcus, lingual gyrus, V1 and V2 (similarly to the AN group), and a cluster in the left premotor cortex, and middle and supplementary frontal gyri. The antisaccade cue > prosaccade cue contrast resulted in three significant clusters: a cluster covering the left middle frontal gyrus, premotor cortex, precentral gyrus, and superior and medial forntal gyri; a cluster covering the right superior frontal gyrus, premotor cortex, superior, middle and medial frontal gyri, SMA and precentral gyrus; and a cluster in the right superior parietal lobule. The prosaccade cue > no-go cue contrast resulted in one significant cluster in the bilateral cuneus, calcarine sulcus, lingual gyrus, V1, V2, associative visual cortex, posterior cingulate cortex, precuneus, parahippocampal gyrus and middle occipital gyrus.

**Table 7:** Significant within groups contrasts for the Prosaccade/Antisaccade/No-Go task for control participants.

Contrast	No. of voxels	Peak *t*	Peak MNI coordinates			Peak regions
			x	y	z	
*Cue period*						
Antisaccade > no-go	1797	7.84	16	-76	6	Cuneus, calcarine
						sulcus, lingual gyrus,
						V1, V2
	163	6.80	-24	-10	56	Premotor cortex,
						middle &
						supplementary
						frontal gyri
Antisaccade > prosaccade	805	10.22	-26	-10	56	Middle frontal gyrus,
						premotor cortex,
						precentral gyrus,
						superior & middle
						frontal gyri
	1198	8.98	24	-6	60	Superior frontal
						gyrus, premotor
						cortex, superior,
						middle & medial
						frontal gyri, SMA,
						precentral gyrus
	255	5.99	30	-54	52	Superior parietal
						lobule
Prosaccade > no-go	5093	9.66	-8	-80	8	Cuneus, calcarine
						sulcus, lingual
						gyrus, V1, V2,
						associative visual
						cortex, posterior
						cingulate cortex,
						precuneus,
						parahippocampal
						gyrus, middle
						occipital gyrus
*Response period*						
Antisaccade > no-go	583	8.95	-10	4	54	Premotor cortex,
						middle & medial
						frontal gyri
Antisaccade > prosaccade	1113	8.19	28	-10	32	Cingulate cortex
Prosaccade > no-go	269	7.26	30	-10	34	Cingulate cortex

Note: V1 = primary visual cortex; V2 = secondary visual cortex; SMA = supplementary motor area; MNI = Montreal Neuroimaging Institute

The antisaccade response > no-go response contrast revealed a significant cluster in the left premotor cortex, and middle and medial frontal gyri. The antisaccade response > prosaccade response contrasts and the prosaccade response > no-go response contrasts both resulted in significant clusters in the right cingulate cortex.

*Mixed design analysis*: The analysis did not result in a significant group x condition interaction (*F*(5, 220) = 8.175, *p*>.05 (FWE)). Simple effects between groups for each contrast did not reveal any significant activations for AN or control participants.

## Discussion

Overall, the findings largely suggest that individuals with AN do not display major saccadic eye movement abnormalities, unlike a number of other psychiatric conditions that are frequently reported to show poor performance on similar saccadic eye movement task batteries, particularly the antisaccade task. The finding of unimpaired antisaccade performance suggests intact functioning in areas of the brain related to volitional saccade control such as the FEF, SEF, DLPFC and ACC. The lack of group differences between AN and HCs in terms of BOLD activity, further supports this conclusion. The AN group did, however, differ significantly in prosaccade performance, evidenced by reduced prosaccade latencies. This finding differs to that commonly reported in other conditions whose prosaccade performance, including prosaccade latencies, is typically unimpaired. However, one study has reported shorter prosaccade latencies in antipsychotic-naïve first-episode schizophrenia patients, which normalised with atypical antipsychotic use [29]. The study by Reilly et al. [29] however differs from several studies which have suggested preserved prosaccade latencies in schizophrenia and other psychiatric conditions, including antipsychotic-naïve patients (e.g. [30,31]). As our group of AN participants were taking a variety of medications, this has the potential to have influenced the findings. However, this finding of shorter prosaccade latencies may be related to the functioning of the superior colliculus (SC). The SC is specifically involved in the initiation and inhibition of saccades. Excitatory inputs from the rostral SC project to the omnipause neurones inhibiting excitatory burst neurons, giving rise to saccades [32]. The SC is involved in triggering saccades and lesions of the SC result in increased prosaccadic latency [33]. However, the injection of gamma-aminobutyric acid (GABA) into the rostral SC has been found to result in shorter latencies to visual targets [34]. Therefore, increased GABA activity in the rostral SC may be involved in the faster prosaccadic latencies observed in AN. Additionally, the shorter latency of prosaccades, but intact latency of antisaccades, suggests a faster neural response in AN than healthy individuals to more reflexive responses requiring fewer resources from higher cognitive areas. Unfortunately, the current dataset cannot interrogate GABA activity in the SC and future magnetic resonance spectroscopy or positron emission tomography work is recommended.

Contrary to our predictions, both antisaccade and memory-guided saccade latency and gain did not differ between groups, suggesting intact visuospatial memory of simple stimuli in AN. AN participants did, however, show an increased rate of inhibitory errors on the memory-guided saccade task. These errors appeared to occur more often with shorter latencies, suggesting participants were generating more reflexive saccades toward the peripheral stimulus rather than an increased rate of inhibitory responses during the response period. Therefore, similarly to the lack of group difference in antisaccade error rate, AN participants did not show a failure to inhibit a planned response in this task. They did, however, demonstrate a failure to inhibit a reflexive response to the peripheral stimulus, which may be indicative of an inhibitory failure during fixation when two competing stimuli are presented. The rate of inhibitory errors was also to a specific target amplitude. Inhibitory errors were significantly more frequent to targets presented at 10°, but not 5°. This provides further support for the hypothesis of GABA and SC involvement as cells that produce saccades are organised topographically in the SC, with smaller amplitude saccades being initiated by more rostral areas and larger saccades by more caudal areas of the SC (Sparks, 2002). Further to this, the injection of the GABA agonist muscimol into the rostral SC, thereby increasing GABA activity, has also been found to increase the rate of inhibitory saccades during a memory-guided saccade task (Munoz & Wurtz, 1993b). Thus, increased GABA in specific regions of the rostral SC may result in the inhibitory problems to this specific amplitude in AN. Similarly to the current study, individuals with OCD have also been found to make more inhibitory errors on a memory-guided saccade task, but only to targets presented at 9° (Rosenberg, Averbach, et al., 1997), suggesting potential involvement of a similar neural mechanism in OCD and AN. Unlike the current study however, the study by Rosenberg et al. [13] did not present stimuli at smaller amplitudes. Therefore, whether intact performance on 5° saccades is also present in OCD has not been evaluated. It would therefore be of interest in future research to not only compare OCD patients with AN on such tasks, but also to utilise a variety of target amplitudes to further interrogate this.

The potential role of altered GABA and the SC in AN is further supported by a recent finding in the same group of participants [35]. In that study, the AN group made a greater number of square wave jerks (SWJs) during fixation. As the injection of GABA into the SC has been found to result in difficulty maintaining fixation, resulting in the production of both microsaccades [36] and unwanted saccades [34], these results provide further support the potential role of GABA and the SC in AN. Furthermore, this rate of SWJs was also found to negatively correlate with state anxiety in the AN group. As anxiety is associated with GABA levels, this finding lends additional support to the hypothesis of increased GABA levels of the rostral SC in AN.

Though the findings of the current study and our previous findings suggest a potential role of GABA and the SC in AN, their roles remain speculative as they were not specifically investigated. Future research utilising magnetic resonance spectroscopy to investigate GABA concentrations in the SC would increase the value of these findings and may assist in the explanation of the results. Furthermore, the utilisation of an enhanced fMRI acquisition sequence may increase the power of detecting activity related to saccadic eye movements. Although the existing literature and our findings provide evidence for the potential role of the SC, other areas such as the frontal eye fields (FEF) and substantia nigra pars reticulata (SNpr), which are also involved in the inhibition of reflexive eye movements, may also be involved. Thus, the interrogation of the entire saccadic system in AN would be of benefit to investigate whether loss of inhibition from SNpr contributes to the possible dysfunction of the SC, or if higher order areas such as the FEF are involved. It would also be beneficial to utilise neuroimaging techniques with superior temporal resolution to fMRI, such as magnetoencephalography, to investigate whether temporal characteristics differ between groups on these tasks; particularly the prosaccade task where reduced latencies were found in the AN group. A further limitation of the study is that AN patients were not medication-free. However, medications that patients were taking, such as benzodiazepines and antipsychotics, are not typically reported to result in reduced prosaccade latencies and increased memory-guided saccade errors as found in the current study (e.g. [30,31,37]). Indeed, benzodiazepines have been found to *increase* saccadic latency [37], whereas antipsychotics are typically found to reduce peak saccadic velocity (e.g. [38]); neither of which were found in the current study.

Overall, this study suggests that individuals with AN have intact performance on a number of saccade tasks that are impaired in other psychiatric populations. The findings are consonant with some reported findings in OCD, particularly an increased rate of inhibitory memory-guided saccade errors of similar amplitude. This increased rate of inhibitory errors, together with the finding of shorter prosaccade latencies in the AN group and our previously reported increase in SWJs, suggest reduced fixation engagement, and a potential role of GABA and the SC in the psychopathology of AN; though, further research is required to interrogate the potential role of other regions of the saccadic system to the findings.
